# Spontaneous Resolution of Subretinal Hemorrhage Secondary to Choroidal Osteoma Unassociated with Choroidal Neovascularization

**DOI:** 10.1155/2014/823953

**Published:** 2014-06-26

**Authors:** Mehmet Talay Koylu, Gokcen Gokce, Yusuf Uysal, Ali Hakan Durukan

**Affiliations:** ^1^Tatvan Military Hospital, Ophthalmology Clinic, 13200 Bitlis, Turkey; ^2^Sarıkamıs Military Hospital, Ophthalmology Clinic, 36500 Sarıkamıs, Turkey; ^3^Gulhane Military Medical School, Ophthalmology Clinic, 06010 Ankara, Turkey

## Abstract

Choroidal osteoma is a rare benign intraocular tumor composed of calcification throughout the choroid. Various treatment modalities are available according to location of the tumor and the cause of the visual distortion. We report herein a 30-year-old male who was referred to our hospital with acute blurred vision as a result of the subretinal hemorrhage from choroidal osteoma. We ruled out the presence of CNV and observation was preferred and we prevented unnecessary treatment attempts as spontaneous recovery is the easiest and safest way.

## 1. Introduction

Choroidal osteoma (CO) which was first described by Gass et al. in 1978 is a rare benign tumor composed of mature bone in the choroid mostly affecting women in their second or third decades [1, 2]. It is generally characterized with unilateral, solitary, yellow-white, or orange coloured choroidal mass with well-defined margins in the peripapillary or macular area [1, 2]. Although most cases are asymptomatic; metamorphopsia, blurred vision, and visual field defects may be initial symptoms [1, 2]. Although the prognosis of the tumor is favourable, vision may be deteriorated by presence of subretinal fluid (SRF), subretinal hemorrhage associated with or without choroidal neovascularization (CNV) [3, 4]. Different treatment modalities including argon laser photocoagulation, photodynamic therapy, transpupillary thermotherapy, intravitreal vascular endothelial growth factor (VEGF) inhibitors, and observation have been suggested to date [4, 5]. We report herein a 30-year-old male with subretinal hemorrhage due to CO unassociated with CNV who recovered without any treatment.

## 2. Case Report

**Figure 1:**
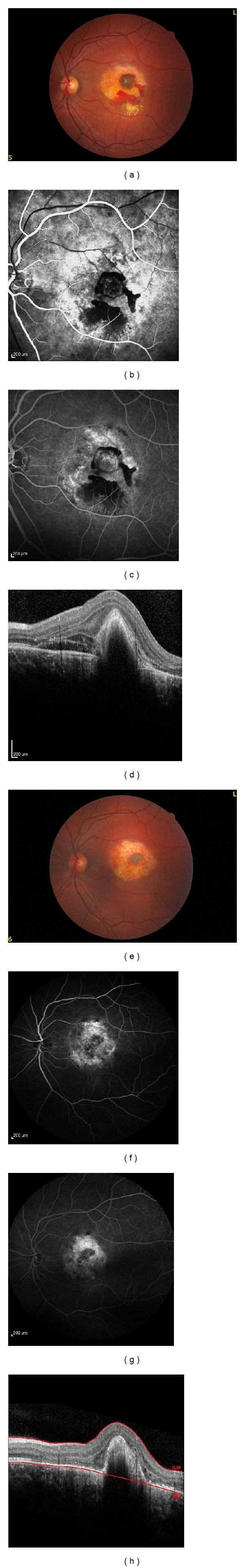
(a) Fundus photography shows subretinal mass with central depigmented area surrounded with subretinal hemorrhage. (b) FFA shows hypofluorescence corresponding to hemorrhage areas, hyperfluorescence due to retinal pigment epithelial window defects in early phase. (c) There is not any staining pattern in late phase. (d) OCT shows high reflectivity associated with dense lesion and subretinal fluid. Two-year follow-up; (e) fundus photography shows no hemorrhage. (f, g) FFA supported the absence of CNV. (h) OCT shows resolution of SRF.

A 30-year-old male was referred to our department with sudden decreased visual acuity in his left eye during watching television 3 days ago. He had no history of systemic or ocular disease. His best-corrected visual acuity (BCVA) was 20/20 in the right eye and counting fingers in the left eye. Fundus examination of the left eye showed subretinal orange and yellow mass measuring three disc diameters with central depigmented area measuring one disc diameter surrounded with subretinal hemorrhage and superficial hemorrhage and hard exudates inferior to lesion (Figure 1(a)). Fundus fluorescein angiography (FFA) revealed hypofluorescence corresponding to hemorrhage areas, hyperfluorescence due to retinal pigment epithelial window defects in early phase (Figure 1(b)), and there were not any late staining indicating the absence of CNV (Figure 1(c)). Optical coherence tomography (OCT) showed high reflectivity associated with dense lesion and SRF fluid at the tumor area (Figure 1(d)). B-scan ultrasonography demonstrated intense reflectivity from the lesion and acoustic shadowing behind. Computed tomography (CT) scan revealed a focal area of calcification involving the choroid. A diagnosis of CO with subretinal hemorrhage without CNV was made. On follow-up examinations, as the hemorrhage disappeared (Figure 1(e)), FFA supported the absence of CNV (Figures 1(f) and 1(g)), SRF resolved, foveal thickness decreased to 252 *μ* from 480 *μ* (Figure 1(h)), and BCVA of the left eye reached 20/32 at 2 years without any treatment.

## 3. Discussion

Choroidal osteoma is a benign choroidal tumor which contains mature bone [4]. The prognosis of CO differs according to tumor localization, presence of CNV, SRF, subretinal hemorrhage, tumor decalcification, and retinal pigment epithelium disturbance [4, 6]. Asymptomatic or extrafoveal osteomas may be observed periodically [7, 8]. Stimulating decalcification with photodynamic therapy and protecting the foveola from tumor invasion is another choice [7, 8]. Secondary CNV may be treated with argon laser photocoagulation, photodynamic therapy, transpupillary thermotherapy, and intravitreal VEGF inhibitors but tumors with subfoveolar involvement of such strategies have a risk for retinal damage and reduced vision [4, 8].

Although Song et al. reported successful results for the treatment of serous retinal detachment secondary to CO in the absence of CNV with VEGF inhibitors, subretinal hemorrhage secondary to CO in the absence of CNV constitutes a challenging issue [4]. As serous retinal detachment and hemorrhage without CNV may resolve spontaneously, it is recommended firstly to observe such episodes [9].

In our case, CNV was ruled out angiographically and clinically and we preferred meticulous follow-up instead of invasive treatments and recommended the patient rest for subretinal hemorrhage and SRF. After two-year follow-up, the hemorrhage and subretinal fluid resolved completely.

The etiology of the subretinal hemorrhage is reported as a result of increased intravascular pressure caused by valsalva maneuvers [3]. To our knowledge, this is the first case of spontaneous subretinal hemorrhage of CO without CNV unassociated with valsalva. In our case, the hemorrhage might be as a result of spontaneous rupture of the choroidal vessels which was distorted by the lesion.

We presented herein a rare condition of spontaneous resolution of subretinal hemorrhage due to CO unassociated with CNV. Clinicians should rule out the presence of CNV in secondary subretinal hemorrhage of CO and prevent unnecessary treatment attempts in the first step as spontaneous recovery is the easiest and safest way.
